# Hebbian and Homeostatic Synaptic Plasticity—Do Alterations of One Reflect Enhancement of the Other?

**DOI:** 10.3389/fncel.2020.00050

**Published:** 2020-03-18

**Authors:** Christos Galanis, Andreas Vlachos

**Affiliations:** ^1^Department of Neuroanatomy, Institute of Anatomy and Cell Biology, Faculty of Medicine, University of Freiburg, Freiburg, Germany; ^2^Faculty of Biology, University of Freiburg, Freiburg, Germany; ^3^Center for Basics in Neuromodulation (NeuroModulBasics), Faculty of Medicine, University of Freiburg, Freiburg, Germany

**Keywords:** hebbian plasticity, homeostatic plasticity, synaptic scaling, amyloid precursor protein, BACE1, APPsα, amyloid-β

## Abstract

During the past 50 years, the cellular and molecular mechanisms of synaptic plasticity have been studied in great detail. A plethora of signaling pathways have been identified that account for synaptic changes based on positive and negative feedback mechanisms. Yet, the biological significance of Hebbian synaptic plasticity (= positive feedback) and homeostatic synaptic plasticity (= negative feedback) remains a matter of debate. Specifically, it is unclear how these opposing forms of plasticity, which share common downstream mechanisms, operate in the same networks, neurons, and synapses. Based on the observation that rapid and input-specific homeostatic mechanisms exist, we here discuss a model that is based on signaling pathways that may adjust a balance between Hebbian and homeostatic synaptic plasticity. Hence, “alterations” in Hebbian plasticity may, in fact, resemble “enhanced” homeostasis, which rapidly returns synaptic strength to baseline. In turn, long-lasting experience-dependent synaptic changes may require attenuation of homeostatic mechanisms or the adjustment of homeostatic setpoints at the single-synapse level. In this context, we propose a role for the proteolytic processing of the amyloid precursor protein (APP) in setting a balance between the ability of neurons to express Hebbian and homeostatic synaptic plasticity.

## Introduction

The ability of neural tissue to adapt to specific stimuli through structural, functional and molecular changes plays a fundamental role in complex brain functions such as perception, decision-making, learning and memory (Citri and Malenka, 2008; Bailey et al., 2015). During the past 50 years, considerable effort has been spent to decipher and better understand the cellular and molecular mechanisms of Hebbian synaptic plasticity, which accounts for activity-dependent changes of synaptic weights based on positive feedback mechanisms (Hebb, 1949; Bliss and Lomo, 1973). It is now well-established that Hebbian plasticity resembles fast and lasting input-specific synaptic changes necessary for experience-dependent memory and learning (Bear, 1996; Chen and Tonegawa, 1997; Klintsova and Greenough, 1999). Experimentally, Hebbian mechanisms have been described in detail for excitatory pre- and postsynaptic sites (e.g., Petzoldt et al., 2016; Monday et al., 2018; Scheefhals and MacGillavry, 2018; Buonarati et al., 2019), where, for example, tetanic electrical stimulation at different frequencies results in the strengthening (long-term potentiation, LTP) or weakening (long-term depression, LTD) of neurotransmission (Bliss and Lomo, 1973; Dudek and Bear, 1992). Meanwhile, evidence has started to emerge for corresponding activity-dependent synaptic changes at GABAergic synapses (Bartos et al., 2011; Rozov et al., 2017; Chiu et al., 2019). Specifically, the plasticity of inhibitory neurotransmission seems to control the ability of neurons to express Hebbian plasticity of excitatory neurotransmission (Letzkus et al., 2015; Zhao et al., 2017).

While feedforward and feedback microcircuits dynamically match afferent excitation to recruited inhibition (Sprekeler, 2017), it has been recognized that, in the absence of physiological constraints, complex systems based solely on positive feedback mechanisms will experience instability—e.g., strong synapses will continue growing, while weakening of synapses will result in synapse elimination (Miller and Mackay, 1994). Indeed, during the past two decades, a plethora of cellular and molecular mechanisms have been identified that maintain neurons in a dynamic functional range by adjusting excitatory and inhibitory synaptic strength in a compensatory manner—i.e., based on negative feedback (Davis and Bezprozvanny, 2001; Marder and Prinz, 2003; Turrigiano and Nelson, 2004; Pozo and Goda, 2010; Keck et al., 2017). Yet, a major unresolved issue in the field concerns the interplay between Hebbian and compensatory—i.e., homeostatic—synaptic plasticity, which share common downstream mechanisms that change and/or adjust excitatory and inhibitory neurotransmission (Turrigiano et al., 1998; Feldman, 2002; Turrigiano and Nelson, 2004; Swanwick et al., 2006; Rannals and Kapur, 2011). Moreover, the biological significance of alterations in Hebbian and/or homeostatic plasticity for pathological brain states remains unclear.

In recent years, these questions have been discussed extensively by leading experts in the field (e.g., Vitureira and Goda, 2013; Fox and Stryker, 2017; Keck et al., 2017; Yee et al., 2017). It has been proposed, for example, that homeostatic plasticity operates on a longer time scale (Turrigiano, 2012; Tononi and Cirelli, 2014; Hengen et al., 2016)—thus not interfering with synaptic changes induced by Hebbian plasticity—and that all synapses of a neuron are adjusted by the same factor in the context of homeostatic “synaptic scaling” to preserve the relative differences between synapses (Turrigiano et al., 1998; Turrigiano, 2008; Vitureira and Goda, 2013). Meanwhile, theoretical modeling work has emphasized the importance of fast homeostatic mechanisms for network stability (Zenke et al., 2013), and robust experimental evidence has been provided for rapid homeostatic plasticity (Keck et al., 2011; Frank, 2014; Li et al., 2014). Furthermore, solid evidence suggests that homeostatic synaptic adaptation can occur locally, in subsets of synapses (e.g., Desai et al., 2002; Kim and Tsien, 2008; Vlachos et al., 2013). These findings indicate that Hebbian and homeostatic synaptic mechanisms may operate in parallel and could thus interfere with each other in the same subset of synapses.

**Figure 1:**
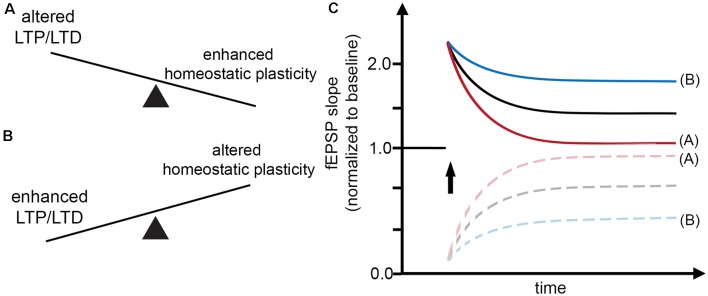
Interaction between Hebbian and homeostatic synaptic plasticity.** (A,B)** Factors may exist which rapidly set a balance between Hebbian and homeostatic synaptic plasticity, thereby affecting the induction and persistence of experience-dependent synaptic changes. **(C)** Alterations in Hebbian plasticity—i.e., long-term potentiation (LTP) or depression (LTD) of evoked field excitatory postsynaptic potentials (fEPSPs; red curve)—may reflect enhanced homeostatic synaptic plasticity. In turn, alterations in homeostatic synaptic plasticity may account for enhanced LTP/LTD (blue curve).

**Figure 2:**
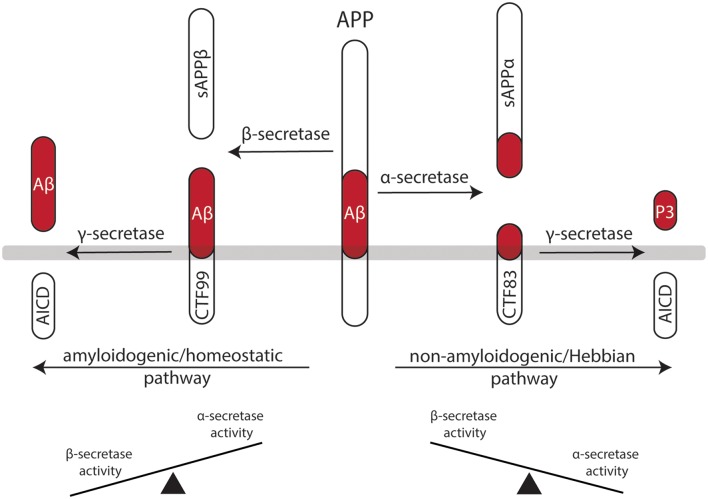
Processing of the amyloid precursor protein (APP) may set a balance between Hebbian and homeostatic synaptic plasticity. Work in recent years has established a firm link between the non-amyloidogenic processing pathway—i.e., APP secreted ectodomain alpha (APPsα)—and the ability of neurons to express LTP of excitatory postsynapses. Likewise, evidence has started to emerge for the role of the amyloidogenic processing pathway—i.e., amyloid-β (Aβ)—in homeostatic synaptic plasticity. Hence, differential processing of APP *via* α- or β-secretases may set a balance between Hebbian and homeostatic synaptic plasticity in neural networks.

In light of these considerations, it is interesting to note that the effects of classic Hebbian plasticity paradigms—e.g., local tetanic electrical stimulation (Bliss and Lomo, 1973)—have not yet been systematically evaluated for their effects on homeostatic synaptic plasticity induction. Therefore, in this article, we sought to present a “*homeostatic view on classic LTP/LTD experiments*” by highlighting mechanisms which may rapidly affect—and hence set a balance between—Hebbian and homeostatic synaptic plasticity (Figure 1). These considerations are put into clinical perspective by discussing the potential role of α- and β-secretase-mediated processing of the amyloid precursor protein (APP) in Hebbian and homeostatic synaptic plasticity (Figure 2).

## Opposing Roles of Ca^2+^ Signaling in Hebbian and Homeostatic Synaptic Plasticity

Central mechanisms that regulate the activity-dependent strengthening (or dampening) of excitatory neurotransmission are modification, trafficking and synthesis of α-amino-3-hydroxy-5-methyl-4-isoxazolepropionic acid receptors (AMPA-Rs) at excitatory postsynapses (Malinow and Malenka, 2002; Diering and Huganir, 2018). Interestingly, both Hebbian and homeostatic synaptic plasticity recruit Ca^2+^-dependent signaling pathways which lead to characteristic changes in synaptic AMPA-R content and function (Malinow and Malenka, 2002; Song and Huganir, 2002; Derkach et al., 2007; Turrigiano, 2008). However, Ca^2+^ influx *via* N-methyl-D-aspartate receptors (NMDA-Rs) or voltage-gated Ca^2+^ channels (VGCCs) can have opposing effects on postsynaptic AMPA-R content in the context of Hebbian and homeostatic synaptic plasticity (Lee et al., 2000; Diering et al., 2014; Diering and Huganir, 2018).

In the case of LTP induction, for example, tetanic electrical stimulation, which triggers Ca^2+^ influx, can lead to an increase in postsynaptic AMPA-R content and hence potentiation of excitatory neurotransmission (= positive feedback mechanism). Conversely, increased intracellular Ca^2+^ levels are expected to trigger homeostatic synaptic down-scaling, which returns AMPA-R content to baseline (= negative feedback mechanism). Considering such rapid interactions between Hebbian and homeostatic plasticity mechanisms (Figure 1), a widely used interpretation of “alterations” in Hebbian plasticity—i.e., failure to persistently change the amplitude or the slope of evoked field excitatory postsynaptic potentials (fEPSPs)—may, in fact, resemble “enhanced” homeostasis, which effectively returns fEPSPs to baseline after the LTP- or LTD-inducing “network perturbation” (see Figures 1A,C). Conversely, signaling pathways that block homeostasis or change homeostatic setpoints will result in persisting changes of excitatory neurotransmission (Figures 1B,C). We have to concede, however, that molecular signaling pathways that attenuate or adjust local homeostatic plasticity at the level of individual synapses are not well-understood. It is also interesting to speculate in this context that changes in the ability of neurons to express homeostatic plasticity *per se* may suffice to generate Hebbian-like associative plasticity. Indeed, a recent study employed computational modeling to demonstrate associative properties of firing-rate homeostasis in recurrent neuronal networks (Gallinaro and Rotter, 2018).

## Role of Dopamine in Homeostatic Synaptic Plasticity

Based on the above considerations, we recently tested for the role of dopamine in homeostatic synaptic plasticity (Strehl et al., 2018). We reasoned that neuromodulators which promote Hebbian plasticity (Otani et al., 2003; Mu et al., 2011; Sheynikhovich et al., 2013; Broussard et al., 2016) may also act by blocking the ability of neurons to express homeostatic synaptic plasticity. Indeed, we were able to demonstrate that dopamine blocks homeostatic plasticity of excitatory neurotransmission in entorhino-hippocampal tissue cultures (Strehl et al., 2018). Pharmacological activation of D_1/5_ receptors, but not D_2/3_ receptors, mimicked the effects of dopamine on homeostatic plasticity. These findings raise the intriguing possibility that dopamine may act as a permissive factor that promotes Hebbian plasticity, at least in part, by blocking homeostasis. Interestingly, the “anti-homeostatic” effects of dopamine were only observed in immature neurons during early postnatal development (Strehl et al., 2018). Hence, specific factors may exist which adjust homeostatic plasticity in specific cells depending on the state of the neural network. It remains to be shown, however, whether dopamine indeed promotes Hebbian plasticity by attenuating homeostatic plasticity at the level of individual synapses and whether dopamine acts on neurons or glia cells (or both) to assert its differential effects on plasticity. Regardless of these considerations, these results call for a re-evaluation of the available LTP/LTD literature and a systematic assessment of well-known “LTP-/LTD-promoting or -blocking factors” in homeostatic synaptic plasticity. As an example that is of considerable clinical relevance, we here discuss the potential role of APP processing in setting a balance between Hebbian and homeostatic synaptic plasticity.

## The Role of The Amyloid Precursor Protein in Synaptic Plasticity

Work in recent years has established a firm link between APP and structural and functional plasticity (comprehensively reviewed in Müller et al., 2017). These studies are based on experiments using APP-deficient mice, or mice in which the APP gene has been genetically modified (Dawson et al., 1999; Magara et al., 1999; Seabrook et al., 1999; Turner et al., 2003; Herms et al., 2004). Historically, the majority of studies in the field have focused on addressing the role of APP and its cleavage products in Hebbian plasticity. More recently, some evidence has supported its involvement in homeostatic synaptic plasticity (Jang and Chung, 2016; Styr and Slutsky, 2018).

APP is a type I transmembrane protein ubiquitously expressed in all mammalian tissues (Müller-Hill and Beyreuther, 1989; Müller et al., 2017). It is differentially processed by secretases *via* two pathways (Figure 2): the *amyloidogenic processing pathway* generates amyloid-β (Aβ) peptides, which are implicated in the pathogenesis of Alzheimer’s disease (AD), while the *non-amyloidogenic processing pathway* produces the neuroprotective soluble ectodomain APPsα (Turner et al., 2003). In the amyloidogenic pathway, APP is cleaved by β-site APP cleaving enzyme (BACE1), which releases APP soluble fragment beta (APPsβ), followed by γ-secretase processing, which generates Aβ fragments and the APP intracellular domain (AICD; Vassar et al., 1999; Van Der Kant and Goldstein, 2015). In contrast, the non-amyloidogenic processing pathway recruits α-secretases releasing APPsα, again followed by γ-secretases that produce the P3 peptide and AICD (O’Brien and Wong, 2011; Van Der Kant and Goldstein, 2015).

## Role of The Non-Amyloidogenic Pathway in Synaptic Plasticity

APP-deficient mice show alterations in dendritic morphologies and dendritic spine counts (Perez et al., 1997; Lee et al., 2010; Tyan et al., 2012; Weyer et al., 2014). These structural defects have been linked to alterations in LTP and deficits in learning and memory (Dawson et al., 1999; Hick et al., 2015). Interestingly, APPsα rescues several of the deficits of APP^−/−^ animals, while APPsβ does not have such a positive effect on Hebbian plasticity (Ring et al., 2007; Hick et al., 2015). Consistent with this suggestion, enhanced LTP is observed in APPsα-treated acute brain slices prepared from rats (Ishida et al., 1997), and behavioral learning is augmented when mice are injected with APPsα (Meziane et al., 1998). Moreover, pharmacologic inhibition of α-secretase activity impairs LTP in rats, which can be rescued by APPsα (Taylor et al., 2008). This line of evidence suggests that APPsα secretion seems to be activity-dependent—that is, LTP-inducing protocols lead to an increase in APPsα (Nitsch et al., 1992; Fazeli et al., 1994). Therefore, it has been proposed that the non-amyloidogenic processing pathway plays an important role in mediating Hebbian synaptic plasticity (Figure 2). However, it should be clearly stated that APPsα has not yet been tested in the context of homeostatic synaptic plasticity. It thus remains to be shown whether some of the “positive” effects of APPsα on activity-dependent structural and functional plasticity are also mediated by its ability to modulate—i.e., to attenuate—homeostatic plasticity mechanisms.

## Role of The Amyloidogenic Pathway in Synaptic Plasticity

The role of APP processing *via* the amyloidogenic pathway has been studied in detail for its pathogenic role in neurodegeneration (Goldsworthy and Vallence, 2013; Nieweg et al., 2015; Gupta and Goyal, 2016; Chen et al., 2017; Youn et al., 2019). What remains less understood is the physiological role of the amyloidogenic processing pathway and Aβ.

It seems well-established that elevated concentrations of Aβ are “synaptotoxic” by hindering the ability of neurons to express LTP, thereby having detrimental effects on learning and memory (Chiba et al., 2009; Jo et al., 2011; Samidurai et al., 2018). In this context, it has been shown that Aβ interferes with neural Ca^2+^ signaling—i.e., it blocks NMDA-Rs and Ca^2+^/calmodulin-dependent protein kinase II (CamKII; Zhao et al., 2004; Townsend et al., 2007; Gu et al., 2009; but see the work in Opazo et al., 2018, which suggests that Aβ activates CamKII). Similar to APPsα, an increase in synaptic activity and NMDA-R stimulation can also lead to an increase in Aβ production (Kamenetz et al., 2003; Lesné et al., 2005). Thus, it has been proposed that an increase in Aβ may act as a negative feedback mechanism by blocking Hebbian synaptic plasticity. In light of the herein proposed model (Figure 1), Aβ may also act by promoting homeostatic synaptic plasticity (see Figure 1).

Indeed, evidence has started to emerge for a physiological role of Aβ in homeostatic synaptic plasticity. For example, the AMPA-R scaffolding protein PICK1 mediates homeostatic synaptic plasticity (Anggono et al., 2011) and has been linked to Aβ-mediated “alterations” in synaptic plasticity (Alfonso et al., 2014). Similar evidence exists for interaction between Aβ and PSD-95 (Roselli et al., 2005; Sun and Turrigiano, 2011), GKAP (Roselli et al., 2011; Shin et al., 2012), calcineurin (D’Amelio et al., 2011; Kim and Ziff, 2014) and STEP_61_ (Kurup et al., 2010). Finally, BDNF and TNFα, which have been firmly linked to homeostatic synaptic plasticity (Rutherford et al., 1998; Stellwagen and Malenka, 2006; Becker et al., 2015), seem to be dysregulated in the AD brain (Fillit et al., 1991; Phillips et al., 1991). Along this line of evidence, a role for microglia in Aβ-mediated alterations in complex brain function has been suggested (Kitazawa et al., 2004; Hansen et al., 2018; Kinney et al., 2018; Hemonnot et al., 2019). However, it is important to note that the majority of these findings are based on experiments employing transgenic mouse models of AD or high concentrations of Aβ. Hence, direct experimental evidence for a physiological role of APP/Aβ in homeostatic synaptic plasticity is currently missing (Figure 2).

## Clinical Implications and Perspective

Considering the detrimental effects of Aβ in Hebbian synaptic plasticity together with promising results in experiments employing a mouse model that expressed familial mutant APP in the absence of BACE1 (Cai et al., 2001; Luo et al., 2001; Roberds et al., 2001), pharmacologic inhibition of BACE1 has been tested as a potential treatment for the cognitive decline in AD (Yan and Vassar, 2014; Coimbra et al., 2018). Indeed, BACE1 inhibitors successfully lowered Aβ levels detected in the cerebrospinal fluid of AD patients (Kennedy et al., 2016; Egan et al., 2018). However, major clinical trials were discontinued due to a series of adverse effects or no improvement and even accelerated cognitive decline in patients (Coimbra et al., 2018; Egan et al., 2019). On the same note, mice lacking BACE1 showed increased neural excitability and spontaneous seizure activity (Hitt et al., 2010; Hu et al., 2010; Zhu et al., 2018; Vnencak et al., 2019), which have been linked to impaired homeostatic mechanisms (Wondolowski and Dickman, 2013; González et al., 2015). Although it is clear that BACE1 targets several other substrates in the nervous system (Barão et al., 2016), these observations support the notion that some of the adverse effects of clinically used BACE1 inhibitors could be explained by an impairment of Aβ-mediated homeostatic synaptic plasticity.

Hence, it will be important to evaluate the significance of APP processing *via* the amyloidogenic and non-amyloidogenic processing pathways in homeostatic synaptic plasticity. We are confident that a systematic assessment of “pro-homeostatic” effects of Aβ and possible “anti-homeostatic” effects of APPsα will provide new and important insights into the intricate interplay between Hebbian and homeostatic synaptic plasticity. These findings may also be of relevance for the development of new therapeutic strategies in neurological and psychiatric diseases associated with alterations in APP processing or increased Aβ levels.

## Author Contributions

CG and AV wrote this manuscript and prepared the figures.

## Conflict of Interest

The authors declare that the research was conducted in the absence of any commercial or financial relationships that could be construed as a potential conflict of interest.
